# Even obligate symbioses show signs of ecological contingency: Impacts of symbiosis for an invasive stinkbug are mediated by host plant context

**DOI:** 10.1002/ece3.5454

**Published:** 2019-07-24

**Authors:** Jannelle Couret, Lynn Huynh‐Griffin, Ivan Antolic‐Soban, Tarik Salam Acevedo‐Gonzalez, Nicole M. Gerardo

**Affiliations:** ^1^ Department of Biological Sciences University of Rhode Island Kingston RI USA; ^2^ Department of Biology Emory University Atlanta GA USA

**Keywords:** invasive species, kudzu bug, mutualism, plataspid, symbiont

## Abstract

**Abstract:**

Many species interactions are dependent on environmental context, yet the benefits of obligate, mutualistic microbial symbioses to their hosts are typically assumed to be universal across environments. We directly tested this assumption, focusing on the symbiosis between the sap‐feeding insect *Megacopta cribraria* and its primary bacterial symbiont *Candidatus* Ishikawaella capsulata. We assessed host development time, survival, and body size in the presence and absence of the symbiont on two alternative host plants and in the insects' new invasive range. We found that association with the symbiont was critical for host survival to adulthood when reared on either host plant, with few individuals surviving in the absence of symbiosis. Developmental differences between hosts with and without microbial symbionts, however, were mediated by the host plants on which the insects were reared. Our results support the hypothesis that benefits associated with this host–microbe interaction are environmentally contingent, though given that few individuals survive to adulthood without their symbionts, this may have minimal impact on ecological dynamics and current evolutionary trajectories of these partners.

**OPEN RESEARCH BADGES:**

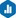

This article has earned an Open Data Badge for making publicly available the digitally‐shareable data necessary to reproduce the reported results. The data is available at https://doi.org/10.5061/dryad.kg4bc56

## 1 INTRODUCTION

Environmental factors have long been shown to mediate the outcome of biotic interactions within and between species (Agrawal, 2001). For example, many studies have examined how environmental context mediates competitive interactions between species (Chesson & Warner, 1981; Hutchinson, 1961; Wiens, 1977). Similarly, the outcomes of many parasitic interactions are contingent upon abiotic (e.g., temperature; Blanford, Jenkins, Read, & Thomas, 2012; Bryner & Rigling, 2011) and biotic (e.g., food availability; Fellous & Koella, 2010; Manson, Otterstatter, & Thomson, 2010) factors. Similar environmental mediation has also been demonstrated in some mutualistic interactions as well (Bronstein, 1994; Piculell, Hoeksema, & Thompson, 2008; Setälä, Rissanen, Markkola, & Setala, 1997; Shan, Lu, Bing, Liu, & Liu, 2014). In the mutualism between leguminous plants and nitrogen‐fixing bacteria housed in root nodules, for example, before root nodule formation is initiated, soil temperature within the root zone influences bacterial survival in the soil as well as the exchange of molecular signals between the two symbiotic partners (Sadowsky, 2005). Nitrogen levels in the soil can also influence these associations; addition of nitrogenous fertilizers reduces bacteria uptake and nodule formation (reviewed in Zahran, 1999). In another plant–microbe association, endophytic fungi in plant tissues have been shown to range from mutualistic to antagonistic toward their plant hosts depending on endophyte genotype, plant genotype, and environmental conditions (Faeth & Fagan, 2002).

The maintenance of mutualistic associations has been described using market theory in which commodity exchanges dictate costs and benefits of the partnership for each “actor” (Werner et al., 2014). However, in the study of “obligate” symbioses, intimate associations between animal or plant hosts and microbial partners that are considered necessary for host survival or reproduction, costs are generally overlooked and benefits to the host are generally assumed to be universal rather than environmentally contingent. Some of the best‐studied models of obligate symbioses are those between insects and bacteria. While little studied, there is some evidence that benefits provided to insects by obligate symbionts may be context‐dependent. Wilkinson, Adams, Minto, and Douglas (2001), for example, demonstrated that aphids with the obligate symbiont *Buchnera aphidicola* had increased larval mass compared to aphids without the symbiont when reared on some host plants but not on others (Wilkinson et al., 2001). In this experiment, however, intimacy of the aphid–*Buchnera* symbiosis required antibiotic clearing of symbionts in mothers, which may have also impacted facultative symbionts and could have introduced other effects. This illustrates the challenge of separating partners in order to quantify the fitness benefits of obligate symbioses for hosts. More recent work, however, which did not involve antibiotic clearance, revealed that aphid genotypes vary in the *Buchnera* titers that they maintain, and suggested that high symbiont titers may come at a cost. This variation would suggest that these coevolved partners are still subject to potential conflict that could select for continued host–symbiont adaptation (Chong & Moran, 2016). Whether the coevolution of such intimate symbioses can be ecologically contingent remains an important question that requires a decoupling of partners across different environments.

Due to the manner of transmission of their obligate symbionts, plataspid stinkbugs of the genus *Megacopta* (Plataspidae) provide a system to quantitatively measure the impacts of obligate symbionts. *Megacopta* spp. harbor the extracellular Gamma‐proteobacteria *Candidatus* Ishikawaella capsulata (hereafter, *Ishikawaella*) in midgut crypts (Fukatsu & Hosokawa, 2002; Hosokawa, Kikuchi, Nikoh, Shimada, & Fukatsu, 2006). The bacteria are vertically transmitted via protein capsules, manufactured by the mother, that are loaded with symbionts and deposited alongside each egg mass (Hosokawa, Kikuchi, & Fukatsu, 2007). Aposymbiotic nymphs hatch and probe these capsules to ingest symbionts (Hosokawa, Kikuchi, Shimada, & Fukatsu, 2008). By removal or heat treatment of symbiont capsules, Fukatsu and Hosokawa (2002) demonstrated that preventing establishment of a symbiont population within *Megacopta punctatissima*, when feeding on soybean in controlled laboratory conditions, can delay development, decrease body weight, and lessen adult coloration, suggesting an essential need for symbiosis for this *Megacopta* species. Notably, this method of disrupting the symbiosis requires no antibiotics, which can have off‐target effects that can influence host phenotypes, in part by altering other components of the host microbiome.

*Megacopta cribraria*, the sister species of *M. punctatissima,* recently invaded North America from the Kyushu region of Japan (Hosokawa, Nikoh, & Fukatsu, 2014). Since being observed in Georgia, United States, in 2009 (Eger et al., 2010), it has expanded its range across the southeastern United States (https://www.kudzubug.org/distribution-map/; Gardner et al., 2013; Leslie et al., 2014; Ruberson et al., 2013). Its expansion is closely associated with the distribution of one host plant, kudzu vine (*Pueraria montana*; Eger et al., 2010; Suiter et al., 2010). In Asia, *Megacopta* spp. occur on kudzu and occasionally on soybean (*Glycine max* (L.) Merril), though reports of the pest status of *M. cribraria* for soybean in Asia are conflicting (Hibino & Itô, 1983; Hosokawa, Kikuchi, Shimada, Kikuchi, Shimada, & Fukatsu, 2007). Studies have shown that Asian *M. cribraria* can more readily feed on soybean when inoculated with the symbionts of *M. punctatissima,* a significant soybean pest (Hosokawa, Kikuchi, Shimada, et al., 2007).

In its expanded North American range, *M. cribraria* is widely reported on soybean (Gardner et al., 2013), suggesting that soybean is a suitable host plant for invasive *M. cribraria* (Blount, Buntin, & Roberts, 2016; Blount, Roberts, et al., 2016; Del Pozo‐Valdivia & Reisig, 2013; Seiter, Greene, & Reay‐Jones, 2013). Adults consume other legumes and some additional angiosperms and conifers as well (Blount, Buntin, & Sparks, 2015; Lovejoy & Johnson, 2014; Medal, Halbert, Cruz, Smith, & Davis, 2016; Medal, Halbert, Smith, & Cruz, 2013). It is presumed that the microbial symbiont *Ishikawaella* is as essential for invasive *M. cribraria* as for its sister species, *M. punctatissima*, but this has not been empirically tested. Moreover, it is not known if the benefits of symbiosis for the host are dependent on environmental context, which has important implications for understanding the biology of this invasive species. Here, we explicitly test the impact of host plant as the environmental context for the host–microbe interaction of *M. cribraria* and *Ishikawaella*. We measure development rate and survival of *M. cribraria* with a normal versus disrupted symbiosis and when reared on alternative host plants in both the laboratory and field. We find that developmental benefits of symbiosis for this host are contingent on the environmental context of host plant, though survival of individuals to adulthood without symbionts is rare, suggesting that the symbiosis is obligate in the invasive range and regardless of the nutrients provisioned by alternative hosts plants.

## 2 MATERIAL AND METHODS

### 2.1 Overview

We measured *M. cribraria* development time, juvenile survival, and adult body size upon emergence in the presence and absence of symbionts when reared on two alternative host plants, leading to a two‐by‐two factorial design of host plant by symbiosis status (normal or disrupted). The first experiment was conducted in the field. We then repeated the design in the laboratory to focus on the development of early instars (hatch to third instar).

### 2.2 Insect sampling and egg mass preparation

**Figure 1:**
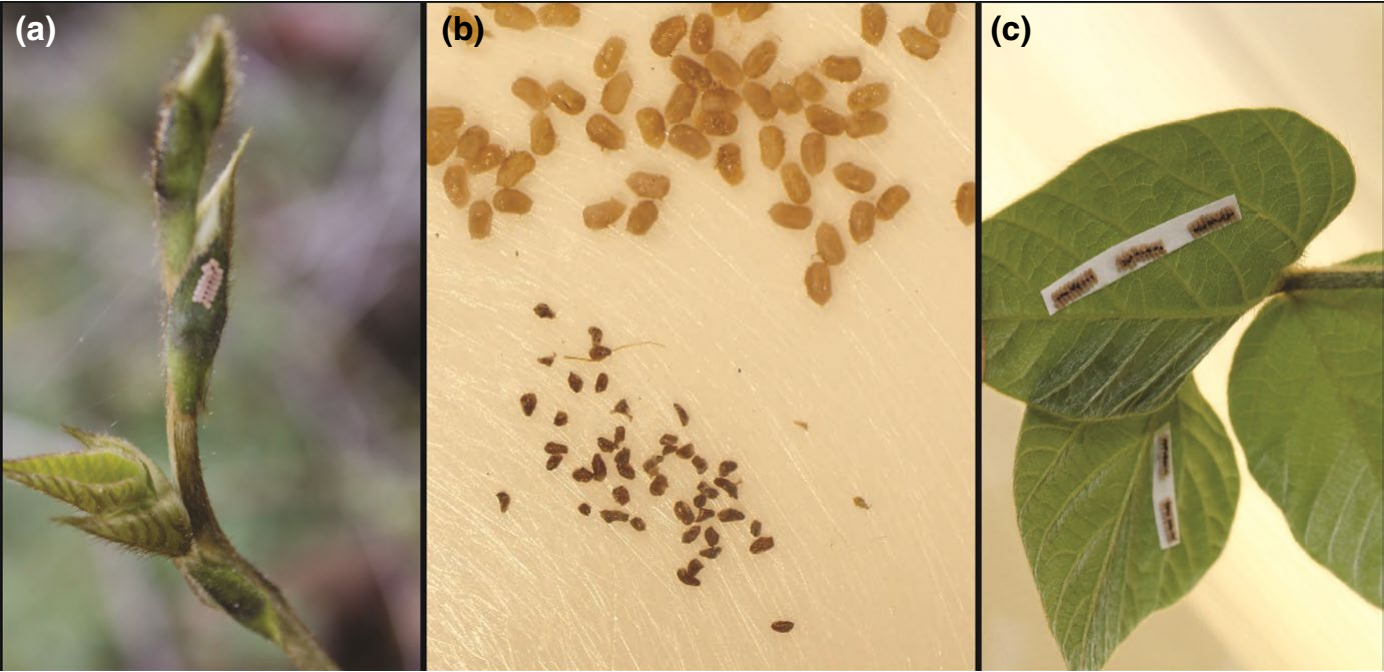
*Megacopta cribraria* egg masses: (a) in natural environment on kudzu, (b) under stereoscopic dissection, and (c) reconstructed into masses and glued to an experimental soybean plant. In (b), the eggs are at the top and the symbiont capsules are at the bottom. When reconstructed (c), the symbiont capsules are between the two rows of eggs as they are laid by females in nature

For both the field and laboratory experiments, we collected egg masses from the top stratum of new kudzu growth on and near the Emory University campus (Atlanta, Georgia, United States). Using forceps, we removed symbiont capsules and separated egg masses into single eggs. We used washes of 70% ethanol and 4% formalin to clean eggs, but not symbiont capsules (Fukatsu & Hosokawa, 2002). We glued eggs together using diluted, nontoxic Elmer's glue into experimental egg masses of 20 eggs, positioned to mimic natural egg mass configurations (Figure 1). To each experimental egg mass, we added 10 symbiont capsules, which were carefully glued along the central line of the experimental egg masses.

We assigned egg masses to either “normal” or “disrupted” symbiosis treatments. In the absence of a symbiont capsule on an egg mass at the time of hatching, *Megacopta* nymphs may wander off host plants (Hosokawa et al., 2008). For the disrupted symbiosis treatment, we therefore provided autoclaved symbiont capsules based on protocols in Fukatsu and Hosokawa (2002), which reduced wandering while preventing establishment of the symbiosis. To suppress symbiont proliferation, we heated symbiont capsules to 100°C for 15 min in an autoclave. Subsequently, we glued autoclaved capsules along the central line of the experimental egg masses for the disrupted symbiosis treatment. For “normal” symbiont treatments, we glued un‐autoclaved symbiont capsules to experimental egg masses.

We sought a sample size of 30 adults for each treatment. We extrapolated the number of eggs needed to achieve this based on the published estimates of *Megacopta* spp. hatch rate (Zhang, Hanula, & Horn, 2012) and juvenile mortality (Fukatsu & Hosokawa, 2002). For all experiments, initial rearing density was 200 eggs/plant with 100 symbiont capsules. We glued egg masses to the underside of leaves.

### 2.3 Experiment setup

We started soybean plants (Public Variety UA 5612) for the field experiment in a greenhouse and transplanted them after two weeks into one of four outdoor raised beds (3 × 6 ft) in a community garden in Doraville, Georgia, United States. Each bed contained 18 plants (one per square foot). Beds were enclosed in a mesh tent (4 × 8 ft) secured to a wooden frame with posts. We used a patch of wild kudzu growing adjacent to the community garden for the kudzu treatment by isolating and enclosing vines. We removed wild *M. cribraria* and other insects from these kudzu shoots manually. We then enclosed shoots in cylindrical mesh tents (2.5 ft long, 18 in diameter). We set up twenty tents one week prior to the beginning of the experiment and checked daily for infiltration of wild *M. cribraria* or other insects. Due to the time constraints of experimental egg mass preparation and delays due to inclement weather, we staggered experimental tent setup in the field over six weeks. Each round of experimental setup included all four treatments, and in total, there were two rounds. Contamination of soy plants with spider mites resulted in the insects reared on 18 soy plants being removed from analysis.

We conducted laboratory rearing experiments on potted plants housed in BudDorm mesh tents (36 × 36 × 72 inches). We potted root crowns from nearby kudzu patches and maintained them in the greenhouse until established (Frye, Hough‐Goldstein, & Sun, 2007; Zhang et al., 2012). We started soybean plants from seed as in the field experiment. Each treatment had two tents, with four potted plants per tent. Plants were evenly spaced such that there was no measurable migration of nymphs from one plant to another. Normal symbiont treatment nymphs did not appear to leave the plants. Some nymphs in the disrupted symbiosis treatment did wander, but any that wandered off of the plant died within a day. We counted all dead nymphs. We recorded information on nymphal development stages by plant and mortality by tent. We housed tents in environmental chambers and maintained them at 25°C, with approximately 70% relative humidity and an extended‐day schedule of 16L:8D.

### 2.4 Life‐history trait measurement

We measured multiple life‐history traits, including development time to each instar, survival to each instar, and adult body size. We determined development time and survival by counting the number of insects and their life stage each week after hatch in the field and each day after hatch in the laboratory. Development time from hatch to each life stage was estimated based on weekly counts. Data collection in the field continued until all insects emerged as adults or died. In the laboratory, data collection continued until all insects matured to third instar. In the laboratory, we removed dead nymphs from tents daily, whereas this was not possible in the field. In both experiments, we counted only insects found on the plant. Especially for early instars in the field, it was not possible/feasible to find insects not on the plants.

We collected adults from the field experiment as they emerged into mesh bags and brought them to the laboratory. We weighed and photographed a subset stereoscopically. We collected approximately equal numbers from each treatment. We measured and compared body size based on the scutellum width of adult insects collected from the field tents. Images were analyzed using ImageJ software to obtain body measurements.

### 2.5 Statistical analysis

We plotted and analyzed survival over time as a step function using Kaplan–Meier survival analysis with the “survival” package in R (Therneau, 2013). We analyzed differences in survival between treatments with the log‐rank test and Cox regression analysis (Klein & Moeschberger, 2003). The end of the field and laboratory experiments corresponded to the time at which insects matured to adulthood and to third instar, respectively.

For the field experiment, we used weekly count data to estimate development time from: hatch to first instar, hatch to second instar, hatch to third instar, hatch to fourth instar, hatch to fifth instar, and hatch to adult. We analyzed these five periods separately as dependent variables. We sought to determine whether the associations between treatment factors and development times were consistent across life stages. We recognize that these variables are overlapping and therefore correlated. While one would ideally compare the time between stadia rather than from hatch to each stage, such data were not estimable based on our counting methodology. We analyzed these six dependent variables using a Poisson distributed generalized linear model (GLM) with a log‐link function. Explanatory factors were host plant species, symbiont status (i.e., normal or disrupted symbiosis), and an interaction term. We used a chi‐square test of the nested models of development time to each life stage to determine the significance of the interaction of symbiont status and host plant. To assess impacts of host plant on body size, we separately assessed the dependent variables of scutellum width and wet weight of adults reared on each plant under the normal symbiosis treatment and assessed the factors of plant and sex using two‐way ANOVA. Because the few individuals that made it to adulthood in the kudzu symbiont‐disrupted treatment were found to be symbiont‐positive (see below), to assess the impact of the symbiosis on body size, we conducted a second two‐way ANOVA using only soybean‐reared adults and the predictors of symbiosis status and sex.

For the laboratory experiment, we monitored development daily and recorded development time from hatch to third instar for each experimental insect. We analyzed these data using a two‐way ANOVA and performed multiple comparisons using Tukey's HSD (honestly significant difference). Factors were host plant and symbiont status. We conducted all statistical analyses in the R statistical programming language v3.0.2 (R Core Team, 2013).

## 3 RESULTS

### 3.1 Impacts of symbiosis and host plant species on development and survival in a field setting

**Figure 2:**
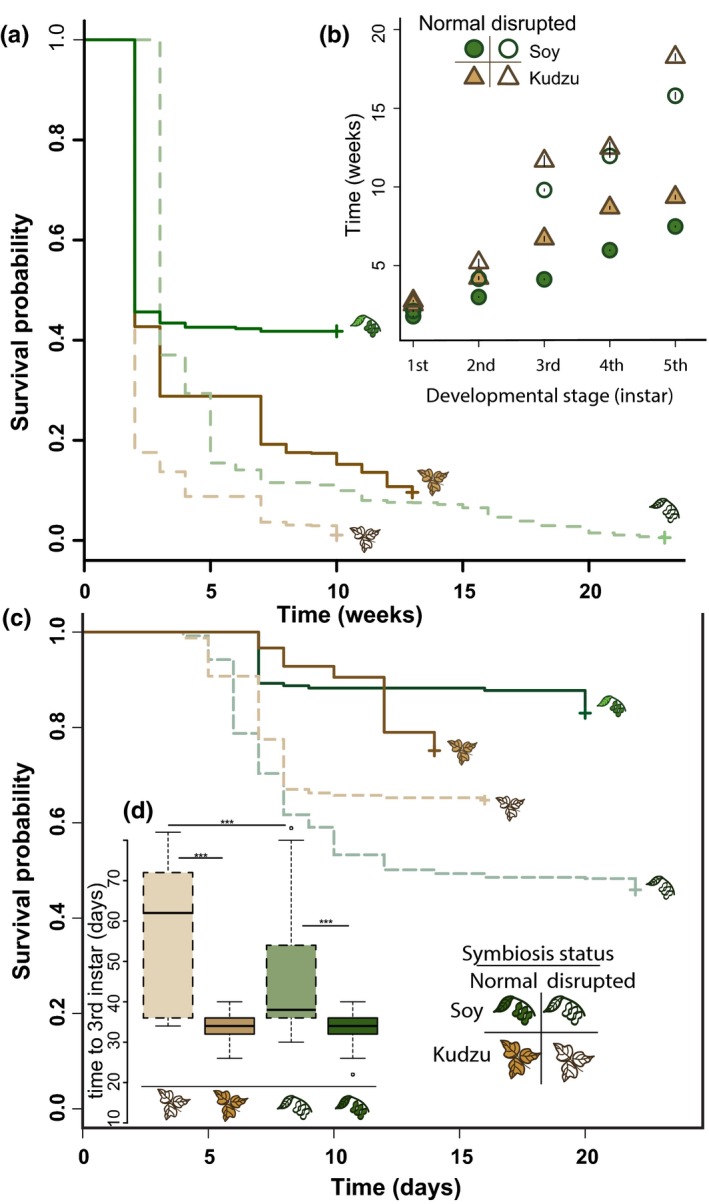
Effects of host plant and symbiosis on *Megacopta cribraria* development time and survival. (a) Survival to adult in the field experiment shown by both host plant and symbiosis status. (b) Development time (in weeks) from hatch to each juvenile life stage in the field experiment. Treatment is indicated by color and line type (see figure legend). Error bars are ±*SEM*. (c) Survival to third instar in laboratory experiment shown by host plant and symbiosis status. (d) Development time in days reared in the laboratory experiment up to the third instar. Panels a, c, and d share a legend shown in panel c

Hatch rates were similar across treatments in the field and ranged from 40% to 50%. Percent survival from hatch to adulthood varied between host plant treatments for the normal symbiosis (41.8% on soy; 9.6% on kudzu). Percent survival was substantially lower in treatments with the disrupted symbiosis (0.6% on soy; 1.1% on kudzu). A log‐rank test indicated significant differences in survivorship between treatments ($\chi_{df=3}^{2}$ = 1,425, *p *< −2^–16^). Survival during the juvenile life stages was lower on kudzu than soy regardless of symbiont status (Figure 2, panel a; Table 1). Host plant, symbiont status, and their interaction were significant predictors of survival during development for *M. cribraria* (Table 2). The greatest mortality occurred in the earliest life stages, namely the first three weeks (Figure 2, panel a).

**Table 1:** Results of Cox regression analysis showing predictors of juvenile survival of *Megacopta cribraria*

	Variable	Coefficient (*B*)	*SE*	*z*	*p* value	Risk ratio	95% CI
Field	Plant	−0.9943	0.03256	−30.537	<.001	0.37	0.3472–0.3943
	Symbiont	−0.6888	0.03629	−18.982	<.001	0.5	0.4677–0.5392
	Plant * Symbiont	0.4072	0.04611	8.832	<.001	1.5	1.3728–1.6448
Laboratory	Plant	−0.4219	0.1116	−3.781	<.001	0.65	0.5270–0.8161
	Symbiont	−1.4231	0.1409	−10.1	<.001	0.24	0.1828–0.3176
	Plant * Symbiont	0.9025	0.1932	4.672	<.001	2.47	1.6886–3.6005

Note

*Plant* is the factor of host plant (kudzu or soybean), and *Symbiont* is the factor of symbiont status (normal or disrupted). The coefficients in the Cox proportional hazard regression represent the increase in the expected log of the relative hazard for each one unit increase in the predictor, holding other predictors constant.

**Table 2:** Results of modeling the development time of *Megacopta cribraria* reared in the field experiment (generalized linear model—GLM) and in the laboratory (two‐way ANOVA)

Life stage	Variable	Coefficient (*B*)	95% CI	*SE*	*z*	*p* value
Field						
First	Intercept	3.09	3.03, 3.15	0.03	104.40	<.0001
	Plant	−0.02	−0.07, 0.04	0.03	−0.55	.586
	Sym	0.70	0.66, 0.75	0.02	31.47	<.0001
	Intercept	3.25	3.16, 3.33	0.04	73.60	<.0001
	Plant	−0.20	−0.29, −0.10	0.05	−4.09	<.0001
	Sym	0.48	0.38, 0.59	0.05	9.06	<.0001
	Plant * Sym	0.26	0.15, 0.38	0.06	4.50	<.0001
Second	Intercept	1.75	1.65, 1.85	0.05	35.38	<.0001
	Plant	1.04	0.94, 1.13	0.05	21.30	<.0001
	Sym	0.38	0.34, 0.43	0.02	16.63	<.0001
	Intercept	1.41	1.21, 1.59	0.09	14.47	<.0001
	Plant	1.39	1.20, 1.59	0.09	14.12	<.0001
	Sym	0.86	0.64, 1.08	0.11	7.75	<.0001
	Plant * Sym	−0.50	−0.73, −0.28	0.11	−4.43	<.0001
Third	Intercept	0.92	0.81, 1.04	0.06	16.12	<.0001
	Plant	1.21	1.10, 1.32	0.06	21.98	<.0001
	Sym	0.80	0.75, 0.85	0.03	30.68	<.0001
	Intercept	1.39	1.22, 1.57	0.09	15.59	<.0001
	Plant	0.72	0.54, 0.90	0.09	7.84	<.0001
	Sym	0.14	−0.08, 0.36	0.11	1.27	.205
	Plant * Sym	0.69	0.47, 0.92	0.11	6.05	<.001
Fourth	Intercept	0.35	0.23, 0.46	0.06	5.96	<.0001
	Plant	1.42	1.32, 1.52	0.05	27.15	<.0001
	Sym	1.54	1.47, 1.60	0.03	47.95	<.0001
	Intercept	1.00	0.76, 1.24	0.12	8.25	<.0001
	Plant	0.74	0.49, 0.99	0.12	5.93	<.0001
	Sym	0.79	0.53, 1.05	0.13	5.91	<.0001
	Plant * Sym	0.78	0.51, 1.05	0.14	5.70	<.0001
Fifth	Intercept	−0.22	−0.39, −0.06	0.08	02.72	<.006
	Plant	1.28	1.16, 1.40	0.06	21.54	<.0001
	Sym	1.94	1.82, 2.06	0.06	31.64	<.0001
	Intercept	1.10	0.70, 1.50	0.20	5.38	<.0001
	Plant	−0.10	−0.52, 0.31	0.21	−0.49	>.6
	Sym	0.56	0.14, 0.97	0.21	2.62	<.008
	Plant * Sym	1.45	1.01, 1.89	0.22	6.54	<.0001
Adult	Intercept	1.99	1.92, 2.08	0.04	51.58	<.0001
	Plant	0.46	0.37, 0.55	0.05	9.92	<.0001

Laboratory						
Third	Plant	1	995	995	12.18	<.0005
	Sym	1	62,471	62,471	764.53	<.000
	Plant * Sym	1	5,138	5,138	62.88	<.000
	Error	191	113,334	0.8		

Note

*Plant* is the factor of host plant (kudzu or soybean), *Sym* is the factor of symbiosis status (normal or disrupted), and *Life stage* is the developmental period from egg hatch to the indicated life stage.

Comparisons of development time to each life stage showed that differences between the normal and disrupted symbiosis treatments widened with each life stage interval (Figure 2, panel b). Results of GLMs of developmental periods from hatch to each life stage indicated that development lagged when reared on kudzu relative to when reared on soybean (Table 2); among only the normal symbiosis treatments across host plant, *M. cribraria* took an average of 1.52 weeks longer to develop on kudzu than on soybean. Furthermore, insects in the disrupted symbiosis treatment showed longer development times than those in the normal symbiosis treatment (Table 2). The interaction between host plant and symbiont status was significant for all development periods (i.e., from hatch to each juvenile instar; Table 2).

**Figure 3:**
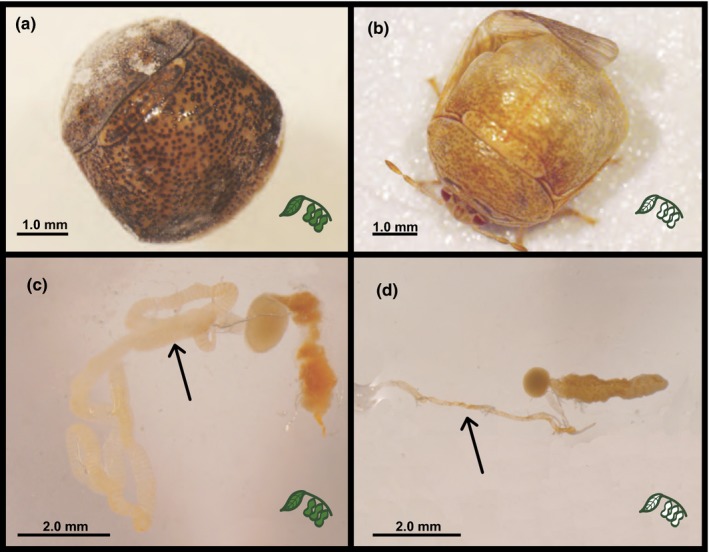
Symbiosis affects cuticle, wing, and gut development. Whole body images (panels a and b) and dissected midgut images (panels c and d, crypt‐bearing midgut sections indicated by arrows) from individuals reared with normal (panels a and c) and disrupted symbiont capsules (panels b and d). All images are from adult female *Megacopta cribraria* reared in the field on soybean

Upon adult emergence, we observed morphological differences in individuals from the normal and disrupted symbiosis treatments for soybean‐reared *M. cribraria* (Figure 3). All individuals from the disrupted symbiosis treatment that made it to adulthood on soybean were pale and had softened cuticles and other abnormalities (Figure 3, panel b), consistent with the symbiont being necessary for proper development and function. Microscopic dissection of these samples revealed stunted midgut development (Figure 3, panel d).

The few disrupted symbiosis subjects that made it to adulthood on kudzu (*n* = 13) did not show these same morphological abnormalities, but these were confirmed to be symbiont‐positive contaminants using qPCR (Hosokawa, Kikuchi, & Fukatsu, 2007). A small proportion of individuals may have acquired symbionts despite the heat deactivation treatment. However, it is more likely, given that they were only found in the kudzu treatment, that this was contamination by nonexperimental insects getting into the kudzu tents, which were of a different form than those used for the soybeans and which were nested within a larger patch of kudzu on which *M. cribraria* were present. This is consistent with the finding that all soybean‐fed putative disrupted symbiosis adults were screened using qPCR and found to be symbiont‐negative. The inclusion of a small number of symbiont‐positive individuals in the kudzu but not soybean measurements does not change our interpretation of the survival and development differences because, if anything, they are decreasing our power to detect differences between the normal symbiosis and disrupted symbiosis kudzu treatments. Furthermore, given that adults are more mobile than nymphs, we believe that these contaminants did not influence our early instar survival and developmental data. We, however, dropped the development time to adulthood and survival to adulthood as variables, instead focusing on other developmental life stages.

**Figure 4:**
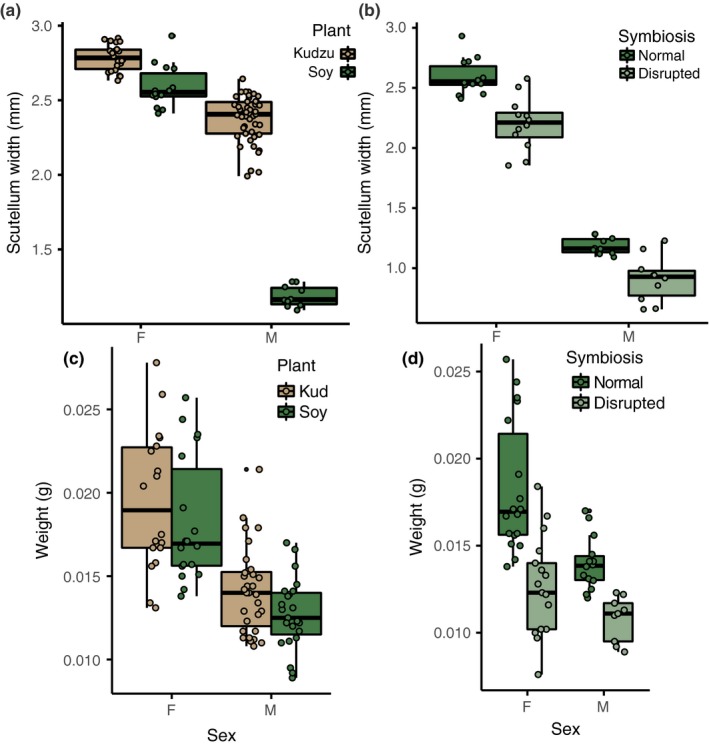
Effect of host plant and symbiosis on adult body size. Scutellum width (mm) of adult *Megacopta cribraria* for normal symbiosis treatments across host plants (panel a). Scutellum width of soybean‐reared insects in normal and disrupted symbiosis treatments (panel b). Wet weight of adult *M. cribraria* across host plants (panel c), and wet weight of normal versus disrupted symbiosis treatments of soybean‐reared insects (panel d)

Among the normal symbiosis treatments, we found significant differences in adult body size as measured by both scutellum width and wet weight between adults reared on soybean and kudzu (Table 3; Figure 4, panels a and c). For both females and males, kudzu‐reared adults were observed to be slightly though significantly larger in body size than those which developed on soybean. A comparison of normal and disrupted treatment groups for soybean‐reared *M. cribraria* indicated that disrupted symbiosis adults had significantly narrower scutellum widths and lower body weights for both males and females. (Table 3; Figure 4, panels b and d).

**Table 3:** Type II ANOVA results for body measurements of adult *Megacopta cribraria* including scutellum width (mm) and wet weight (grams)

Soybean‐reared treatments (normal and disrupted symbioses)							
Scutellum width						mm (*SD*)	
	Sum Sq.	*df*	*F* value	*p *(>*F*)		Normal	Disrupted
Sym	1.32	1	47.35	<.0000	Male	1.19 (0.07)	0.09 (0.19)
Sex	20.58	1	740.09	<.0000	Female	2.59 (0.14)	2.20 (0.22)
Sym:Sex	0.036	1	1.28	.265			
Residuals	1.17	42					

Wet weight						Grams (*SD*)	
	Sum Sq.	*df*	*F* value	*p *(>*F*)		Normal	Disrupted
Sym	0.0003	1	40.99	<.0000	Male	0.014 (0.002)	0.011 (0.001)
Sex	0.0001	1	18.19	<.00009	Female	0.018 (0.004)	0.012 (0.002)
Sym:Sex	0.00002	1	2.76	.102			
Residuals	0.0004	42					

Soybean‐ and kudzu‐reared treatments (normal symbiosis only)							
Scutellum width						mm (*SD*)	
	Sum Sq.	*df*	*F* value	*p *(>*F*)		Kudzu	Soy
Sex	9.86	1	570.39	<.0000	Male	2.37 (0.15)	1.19 (0.07)
Plant	7.76	1	448.49	<.0000	Female	2.78 (0.09)	2.59 (0.14)
Sex:Plant	4.13	1	238.97	<.0000			
Residuals	1.5	87					

Wet weight						Grams (*SD*)	
	Sum Sq.	*df*	*F* value	*p *(>*F*)		Kudzu	Soy
Sex	0.0004	1	31.28	<.0000	Male	0.014 (0.004)	0.013 (0.002)
Plant	0.0018	1	14.41	.00025	Female	0.020 (0.004)	0.016 (0.003)
Sex:Plant	0.00004	1	3.51	.064			
Residuals	0.0013	87					

Note

Comparison of the factor of symbiosis status (normal or disrupted; *Sym*) is shown for soybean‐reared treatments. Comparison across host *Plant* (kudzu or soybean) and sex is shown for only symbiosis‐normal individuals. Data and analysis do not include individuals from symbiosis‐disrupted, kudzu‐reared adults as the few symbiosis‐disrupted individuals that were reared on kudzu were confirmed via qPCR to have symbionts (see Results 2.4).

### 3.2 Impacts of symbiosis and host plant species on development and survival in a laboratory setting

Hatch rates in the laboratory ranged from 70% to 90%, higher than those observed in the field, and were not affected by treatment. There were significant differences in survival to third instar between treatments ($\chi_{df=3}^{2}$ = 142, *p *< −2^–16^). As with the field experiment, host plant, symbiont status, and their interaction were significant predictors of survival during early developmental stages (Table 2), and the significance of the interaction term as a covariate was confirmed using a Wald test, ($\chi_{df=3}^{2}$ = 126.5, *p *< −2^–16^). For treatments with normal symbiont capsules, significant differences in survival between host plant treatments became evident at day 12, with a reduction in survival on kudzu below that of soybean. Disrupted symbiosis treatments showed decreased cumulative survival over time compared to normal symbiont treatments (Figure 2, panel c). The difference in survival probability over time between disrupted and normal symbiosis treatments was wider on soybean than kudzu, a result consistent with the field experiment.

In a pattern consistent with the field results, development time from hatch to third instar was longer for insects reared on kudzu than on soybean. In this setting, *M. cribraria*, across normal and disrupted symbiosis treatments, reared on kudzu took an average of 1.98 days longer to mature to third instar than on soybean. (Tukey's HSD of means, adj. *p* < .0005). This difference is primarily driven by the difference in disrupted treatments, for which on average kudzu‐reared insects took approximately 8.65 days longer to develop to third instar than those on soy (adj. *p *< −2^–16^). For normal symbiosis treatments, the data show only a slight difference (less than 1 day) in the time to develop to third instar between kudzu‐ and soy‐reared *M. cribraria*. Comparing across both host plants, development time was slower for disrupted compared to normal symbiosis treatments (Figure 2, panel d), with a difference of approximately 14.1 days (Tukey difference of means, adj. *p* < .000). The results of a two‐way ANOVA of development time from hatch to third instar indicated that host plant, symbiont status, and the interaction of these factors were significant predictors (Table 2, Figure 2, panel d).

## 4 DISCUSSION

Environmental context can dramatically change when organisms invade a novel habitat, and microbial symbionts can play an important role in successful invasion. The ability of the kudzu vine to invade new habitats, for example, is due in part to its ability to establish symbioses with native mycorrhizal fungi (Greipsson & DiTommaso, 2006), and viruses brought by other invasive plants can aid invasions by weakening native species in competition with invaders (Malmstrom, McCullough, Johnson, Newton, & Borer, 2005). In contrast, the legume *Cytisus scoparius* is limited in its ability to colonize new habitats because it is symbiont‐limited (Parker, Malek, & Parker, 2006). Similarly, in animals, the colonization success of invasive bark beetles depends on its associations with fungi and gut‐associated bacteria (Klepzig, Adams, Handelsman, & Raffa, 2009; Klepzig & Six, 2004; Vasanthakumar et al., 2006). Beyond nutrition, microbial symbionts can also alter other important traits, including heat tolerance (Dunbar, Wilson, Ferguson, & Morales, 2007), parasite and pathogen defense (Oliver, Degnan, Burke, & Moran, 2010), and mating behavior (Miller, Ehrman, & Schneider, 2010), all of which could influence colonization success.

We hypothesized that environmental conditions could alter the beneficial impacts of this symbiosis on host life‐history traits. In both field and laboratory experiments, we found that development time, survival, and body size were significantly impacted by the status of the symbiosis, by the host plant, and, interestingly, by the interaction between these factors. This interaction suggests the overall importance of environmental context in mediating the benefits of symbionts on host fitness, even when those symbionts are obligate partners for the host. The benefits of the association with *Ishikaewella* bacteria for invasive *M. cribraria* across host plants suggest that the symbiosis is vital to the persistence and spread of this invasive pest species.

### 4.1 Effects of host plants on life‐history traits of an invasive pest

Many ecological and environmental factors change for invasive species that could alter host life‐history traits and other phenotypes. The invasion and expansion of *M. cribraria* are closely associated with the distribution of the invasive legume kudzu, their presumed primary host plant in both their native and invasive range (Eger et al., 2010; Suiter et al., 2010). Early on in their North American invasion, however, *M. cribraria* populations were also observed on soybean (Eger et al., 2010), and research indicates that invasive *M. cribraria* can develop on soybean (Ruberson et al., 2013; Seiter et al., 2013). This is surprising because *M. cribraria* is not usually considered a soybean pest in its native range (Hosokawa, Kikuchi, Shimada, et al., 2007). Interestingly, the symbiont genotype of the invasive *M. cribraria* population closely resembles that of the soybean pest symbiont genotype that is associated with *M. punctatissima* in Japan, suggesting that *M. cribraria* may have arrived with the ability to use soybean (Brown, Huynh, Bolender, Nelson, & McCutcheon, 2014). Therefore, we sought to compare critical life‐history traits during development on kudzu, the presumed primary host, and on soybean, an additional host and important agricultural crop with a wide distribution that could facilitate further invasion.

Though kudzu is considered the primary host plant of invasive *M. cribraria*, we surprisingly observed significantly shorter development time and higher juvenile survival on soybean than on kudzu in the field. This difference became apparent in the later instar stages and continued to adult emergence. This suggests that soybean could support substantial growth of *M. cribraria* populations. In the current invasive range, overwintering *M. cribraria* adults generally necessarily oviposit on kudzu, which emerges earlier in the spring than soybean, which is typically planted later in the summer. Evidence suggests, however, that, when available, early‐planted soybean (i.e., planted from April to June) is at greater risk for yield loss due to *M. cribraria* than later‐planted soybean (Blount, Buntin, et al., 2016; Blount, Roberts, et al., 2016). Findings of increased juvenile survival and more rapid development on soybean than on kudzu indicate that early‐planted soybean could fuel rapid population growth of *M. cribraria* that could then carry over for the rest of the season.

Interestingly, despite more rapid development and increased survival on soybean compared to kudzu, *M. cribraria* appear to prefer kudzu over soybean in controlled choice tests (Huskisson, Fogg, Upole, & Zehnder, 2015). Insect preference for host plants is complex, but one possibility is that *M. cribraria*'s counterintuitive preference for kudzu could be driven by benefits to *M. cribraria* upon maturation. Despite improved developmental performance on soybean, we found significantly reduced body size of both males and females reared on soybean compared to those reared on kudzu in the normal symbiosis treatments. In *M. punctatissima*, sexual selection depends on male body size, with significantly higher mating success for large males (Himuro, Hosokawa, & Suzuki, 2006), suggesting that preference for kudzu could be maintained by sexual selection. Furthermore, we found slightly though significantly smaller body size for females reared on soybean, and it is unknown what effects this could have on female fecundity or sexual selection. Though beyond the scope of this study, prediction of plant utilization by invasive *M. cribraria* will require further study of the impact of different plants on other life‐history traits, including oviposition site choice, mate choice, and fecundity.

### 4.2 The role of *Ishikawaella* in *M. cribraria* development

In previous studies, nymphs of sister species *M. punctatissima* exhibited delayed development, alterations in behavior of nymphs, arrested growth, and abnormal body coloration when deprived of symbionts (Fukatsu & Hosokawa, 2002; Hosokawa et al., 2008). We observed similar delayed development and smaller body size in invasive *M. cribraria* as a result of symbiont deprivation. Smaller body sizes were evident for insects with disrupted symbiosis for both sexes, slightly more so for females than males. Furthermore, in the laboratory experiment, recording the wandering behavior of nymphs in days one through nine, we found a difference between the disrupted and normal symbiosis treatments, with disrupted symbiosis individuals wandering off of egg masses and wandering off of the host plants more frequently and earlier on than the normal symbiosis treatments for both soy and kudzu (Figure S1). This finding is consistent with the finding of Hosokawa et al. (2008) examining the impact of symbiont acquisition on the behavior of *M. punctassima* nymphs.

In addition, the few symbiont‐free individuals that made it to adulthood in the field were abnormally pale, similar to the lightening of cuticular pigment induced by partial interference of Laccase2 gene through RNAi in *M. punctatissima* (Futahashi et al., 2011). These results suggest that the symbiont may play a role in the expression of this and potentially other pigmentation and cuticle development genes. Furthermore, symbiont‐deprived adults had strongly reduced gut development (Figure 3), suggesting an important role for the symbiont in mediating critical developmental processes. Taken together, these results indicate that this is an obligate symbiosis for *M. cribraria*.

That *M. cribraria* are dependent on these symbionts on both host plants is consistent with the hypothesis that the bacteria provide critical resources that complement nutrients acquired from feeding on phloem sap. Population genome sequencing of *Ishikawaella* associated with invasive *M. cribraria* indicates that the symbiont genome encodes for most steps critical in the biosynthesis of essential and nonessential amino acids, as well as vitamins, including biotin and riboflavin (Brown et al., 2014). The long‐term coevolutionary history of *Megacopta* hosts and their symbionts (Hosokawa et al., 2006), however, may have made the host dependent on symbiont signaling to mediate other processes as well, and further exploration of the interdependency of these hosts and symbionts will be necessary if disruption of the symbiosis is to be a target of control efforts for this pest (Douglas, 2007).

### 4.3 Benefits of symbiosis and environmental context

Given the challenge of disrupting obligate symbioses, many of which are internally, maternally transmitted, most research on the combined influences of symbiosis and environment are restricted to facultative or horizontally transmitted symbioses. Such studies have shown that symbioses with fungal endophytes and precipitation, for example, interact to influence the ecology of dune‐building grasses (Emery, Bell‐Dereske, & Rudgers, 2015) and that symbiosis with bacteria and plant secondary compounds interact to influence the ecology of pine weevils (Berasategui et al., 2017).

Exploiting the unique mode of *Megacopta* symbiont transmission, we found, both in field and in laboratory settings, that the difference in development time between disrupted and normal symbiosis treatments was larger at each life stage from first to fourth instar in the soybean treatments than the kudzu treatments. For example, in the field, on soybeans, the disrupted symbiosis treatment took approximately 6 weeks longer to develop to fourth instar than the normal symbiosis treatment, compared to a 3.7‐week difference on kudzu. The differences in the adverse impacts of disrupting the symbiosis across host plants demonstrate that even obligate associations can be environmentally contingent. Similar differences across plants in the costs to disrupting symbiosis were observed in the laboratory, although under artificial conditions, the difference between disrupted and normal symbiosis treatments is greater on kudzu than soybean. We attribute this difference to plant health, as kudzu did not grow as well under grow lights in the laboratory as it grew under natural light in the field, but soybean thrived in both field and laboratory conditions.

These differences between treatments and between laboratory and field results underscore the environmental contingency of the benefits of this obligate symbiosis, and there are many potential mechanisms by which this could occur. Environmental contingency in this system may result from the plants providing different nutrients to the insects that in turn differentially complement those provided by the bacteria. As mentioned above, the symbiont genome encodes for most steps critical for biosynthesis of essential and nonessential amino acids, and several vitamins. Focusing on riboflavin (vitamin B_2_) as an example, plant riboflavin biosynthesis differs across species and is regulated by many environmental factors, including light (Smith, Croft, Moulin, & Webb, 2007) and bioavailability of iron (Vorwieger et al., 2007). Such variation in vitamin availability is a potential means by which the plant‐by‐symbiont interaction could influence insect host development.

It is important to note that these differences in the relative benefit of the symbiosis on the two host plants probably have little influence on the evolutionary dynamics of the system at this time, as ultimately the main driver of host fitness is having the symbiosis. However, as these host and symbiont populations accumulate genetic diversity and adapt to their new environment, the ecological contingency of this interaction could become an important factor in the evolutionary trajectory of the system. While host and symbiont populations exhibited little genetic diversity within the first several years of invasion (Brown et al., 2014), as the invasion spreads there is potential for both the insects and their symbionts to diverge and potentially adapt to conditions associated with alternative hosts plants, which could in turn influence the benefits conferred by the symbiosis. Future work should explore this diversification through coupling of continued population genomic analyses and experimental assessment of how genetically diversified invasive hosts and symbionts interact with host plants and other ecological conditions to mediate host phenotypes.

## 5 CONCLUSION

A major obstacle to understanding the importance of obligate symbioses in determining host phenotype and ecology has been the inability to experimentally decouple the host and symbiont. The unique biology of the *M. cribaria* system facilitates this experimental disruption and manipulation of an obligate symbiosis across environmental conditions. Here, the environmental context of host plant can mediate the effect of obligate microbial symbionts on the life history of their animal host. This finding has implications for understanding the ecology, evolution, and invasion potential of *M. cribraria* in its expanded North American range. The developmental performance of *M. cribraria* on different host plants will influence the boundaries of its invasive range in the United States, with potentially important consequences for the agriculture of soybean.

## CONFLICT OF INTEREST

The authors are not aware of any conflict of interest in the publication of this work.

## AUTHOR CONTRIBUTIONS

JC conceived the study, collected and analyzed data, and drafted the manuscript. NMG conceived the study and collected data. LGH collected and analyzed data. IAS collected data. TAG conducted pilot studies and collected data. All authors revised the manuscript.

## DATA AVAILABILITY STATEMENT

Experimental data and all R Code used for statistical analyses are archived in the Dryad data repository: https://doi.org/10.5061/dryad.kg4bc56
