# Supplementary material for: Even obligate symbioses show signs of ecological contingency: Impacts of symbiosis for an invasive stinkbug are mediated by host plant context
Source: Ecol Evol. 2019 Jul 24;9(16):9087–99. doi: 10.1002/ece3.5454 (PMC6706230; doi:10.1002/ece3.5454)
Supplement: Supplementary file 1 [file ECE3-9-9087-s001.docx]

**Appendix**

Even obligate symbioses show signs of ecological contingency: impacts of symbiosis for an invasive stinkbug are mediated by host plant context

**Figure A1.** **Wandering behavior of *M. cribraria* nymphs.** Behavior of nymphs observed from days 1-9 in laboratory experimental tents. On egg mass signifies a lack of movement. Wandering signifies movement separated by location (either on plant or on tent).
